# Charlson comorbidity index is predictive of postoperative clinical outcome after single-level posterior lumbar interbody fusion surgery

**DOI:** 10.1186/s13018-021-02377-7

**Published:** 2021-03-30

**Authors:** Kensuke Shinonara, Ryo Ugawa, Shinya Arataki, Shinnosuke Nakahara, Kazuhiro Takeuchi

**Affiliations:** Okayama Medical Center, Department of Orthopaedic Surgery, National Hospital Organization, 1711-1 Tamasu, Kitaku, Okayama city, Japan

**Keywords:** Charlson comorbidity index, Posterior lumbar interbody fusion, Clinical outcome, Improvement rate, Complication, Length of stay, Direct cost

## Abstract

**Background:**

In several previous studies, Charlson comorbidity index (CCI) score was associated with postoperative complications, mortality, and re-admission. There are few reports about the influence of CCI score on postoperative clinical outcome. The purpose of this study was to investigate the influence of comorbidities as calculated with CCI on postoperative clinical outcomes after PLIF.

**Methods:**

Three hundred sixty-six patients who underwent an elective primary single-level PLIF were included. Postoperative clinical outcome was evaluated with the Japanese Orthopaedic Association lumbar score (JOA score). The correlation coefficient between the CCI score and postoperative improvement in JOA score was investigated. Patients were divided into three groups according to their CCI score (0, 1, and 2+). JOA improvement rate, length of stay (LOS), and direct cost were compared between each group. Postoperative complications were also investigated.

**Results:**

There was a weak negative relationship between CCI score and JOA improvement rate (*r* = − 0.20). LOS and direct cost had almost no correlation with CCI score. The JOA improvement rate of group 0 and group 1 was significantly higher than group 2+. LOS and direct cost were also significantly different between group 0 and group 2+. There were 14 postoperative complications. Adverse postoperative complications were equivalently distributed in each group, and not associated with the number of comorbidities.

**Conclusions:**

A higher CCI score leads to a poor postoperative outcome. The recovery rate of patients with two or more comorbidities was significantly higher than in patients without comorbidities. However, the CCI score did not influence LOS and increased direct costs. The surgeon must take into consideration the patient’s comorbidities when planning a surgical intervention in order to achieve a good clinical outcome.

## Background

Medical technology and spine surgery techniques have progressed rapidly, benefitting patients suffering from spinal disease. The indications for spine surgery have broadened to include the elderly and patients with several medical comorbidities. It is very important to assess the patient’s preoperative general condition and comorbidities to assess surgical safety before making a surgical decision. Campbell et al. [1] reported that an increased number of comorbidities strongly correlated with an increased risk of complications after spine surgery, and concluded that comorbidities significantly increase the risk of perioperative complications. Hence, the quantification and evaluation of comorbidities is required before surgery.

The Charlson comorbidity index (CCI), first reported in 1987, is a useful method for evaluating medical comorbidities [2]. It is a simple, easy, and user-friendly scoring index. The CCI is widely used in various surgical fields as a predictor of mortality or postoperative complication. Sato [3] reported that CCI was an independent predictor of postoperative complications following lung cancer surgery. Similar findings were observed after colon cancer surgery by Huang [4], and following a percutaneous nephrolithotomy by Unsal [5].

Several reports about CCI and orthopedic spine surgery have been published. Voskuijl et al. [6] reported that every point increase in CCI score added an additional 0.9% risk of readmission to patients undergoing spine surgery. Especially in spine surgery, Harris et al. [7] retrospectively studied 640 patients with cervical spine fractures, reporting that higher CCI scores were associated with an increased risk of mortality. A retrospective study of 200 patients who underwent surgery for spinal metastases concluded that CCI score was the most significant predictor of 30-day complications [8].

Posterior lumbar interbody fusion (PLIF) is a well-known and common surgical procedure for degenerative lumbar disease. Surgical interbody fusion of the degenerative lumbar spine can improve the patient’s low back or lower leg pain and disability. Evidence supporting good clinical outcomes after PLIF has already been reported [9–11].

Several reports have previously identified a relationship between CCI and spine surgery [1, 6–8]. However, few have investigated a correlation between CCI and postoperative clinical and functional outcome.

The aim of this study is to determine if the CCI is a predictor of postoperative outcome after PLIF. In addition, this study seeks to investigate correlations between CCI, operative time, intraoperative blood loss, length of stay (LOS), and direct cost of admission. The idea of this study is based translational orthopedics filling the gap between internal medicine and orthopaedic surgery as same as basic science and clinical science [12, 13]. This analysis would be helpful for aiding the surgical decision-making of spine surgeons.

## Methods

This is a clinical retrospective study performed at one institution. Patients who underwent PLIF from 2014 to 2018 were analyzed. Inclusion criteria were all elective single-level primary surgeries. Multilevel surgeries, additional or revision surgeries, and unplanned surgeries were excluded. PLIFs were performed using cages filled with autologous bone from the lamina, spinous process, and facet joints. Posterior instrumentation with pedicle screws was performed in all cases. Drains were removed on postoperative day 3 in all patients. All patients were required to wear a lumbar corset for three months after surgery. All patients were followed as out-patients for at least 1 year.

The medical record of these patients was reviewed and information on CCI score, operative time, intraoperative blood loss, Japanese Orthopaedic Association (JOA) lumbar score with a maximum point of 29, JOA improvement rate, postoperative complications, LOS, and direct cost were collected. CCI score was calculated using medical history as reported by the patient, cited in the medical record, or detected during the medical examination. CCI score was divided into three groups for analysis (0, 1, 2+), reflecting the criteria from previous reports [7]. The JOA improvement rate of each group was calculated and compared for significant differences. A postoperative complication was defined as an adverse event that required additional surgery within 30 days after PLIF.

JOA scores were evaluated based on previous work by Hioki et al. [14]. JOA score was useful for evaluating physical state, functional status, and daily-activity. JOA improvement rate was defined as: (Postoperative JOA score–Preoperative JOA score/29–Preoperative JOA score) × 100(%). Direct cost was exchanged from Yen to U.S Dollars using the rate at the day for 23 June 2020 (1 US dollar = 107 Yen).

The correlation coefficient between CCI score and JOA improvement rate was measured. Additionally, the correlation coefficient between CCI score and LOS or direct cost was also calculated.

Collected data was analyzed using Excel (Microsoft, Redmond, WA) and SPSS for Windows Version 25 (SPSS, Chicago, IL). Several statistical tests (Pearson and Kruskal-Wallis test) were used to calculate a correlation coefficient and significant differences. *P* values less than 0.05 were considered significant.

The institutional review board approved this clinical retrospective study (Okayama Medical Center, Number: 2018-137).

## Results

A total of 366 patients met the inclusion/exclusion criteria and were divided into group 0 (*n* = 137), group 1 (*n* = 101), and group 2+ (*n* = 128). The mean age of all patients was 69 years old. There were 158 males and 208 females. The average follow-up period was 24 months. The mean CCI was 1.3 and diabetes mellitus was the most common comorbidity (Table 1). The mean operative time was 124 min, and the mean intraoperative blood loss was 145 ml. The preoperative and final JOA scores were 16.9 and 25.5, respectively. The mean JOA improvement rate was 72.1%. There were 14 postoperative complications (incident rate 3.8%). Seven patients had a postoperative deep wound infection that required additional surgery. Five patients had a postoperative hematoma that required a return to the operating room. Two patients had implant failures that required the re-insertion of a cage or screws. The number of complications in each group was 4 cases (2.9%) in group 0, 7 cases (6.9%) in group 1 and 3 cases (2.3%) in group 2+. The mean LOS was 21 days and the mean direct cost was 21,192 US dollars.

**Table 1:** Patient number and the incidence of each comorbidity in this study

	All patients = 366	
Comorbidity	Patient number	Incident rate (%)
1 point		
Myocardial infarction	50	13.6
Congestive heart failure	55	15
Peripheral vascular disease	18	4.9
Cerebrovascular disease	30	8.1
Dementia	6	1.6
Chronic pulmonary disease	31	8.4
Connective tissue disease	11	3
Ulcer disease	31	8.4
Mild liver disease	24	6.5
Diabetes	79	21.5
2 points		
Hemiplegia	5	1.3
Moderate or severe renal disease	25	6.8
Diabetes with end organ damage	4	1
Any tumor	33	9
Leukemia	2	0.5
Lymphoma	4	1
3 points		
Moderate or severe liver disease	0	0
6 points		
Metastatic solid tumor	2	0.5
AIDS	0	0

*AIDS* acquired immune deficiency syndrome

**Fig. 1:**
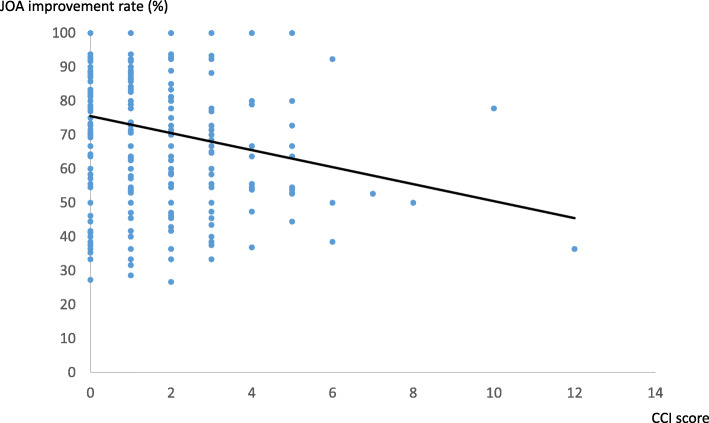
Increasing CCI score negatively correlated with JOA improvement rate. JOA; Japanese Orthopaedic Association, CCI; Charlson comorbidity index

**Fig. 2:**
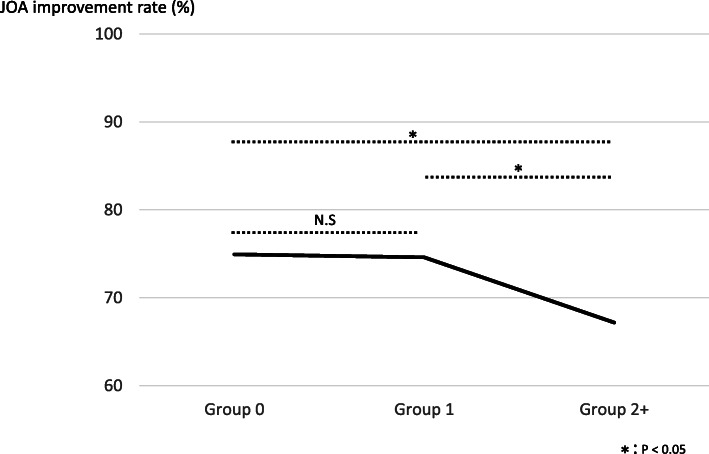
There was no significant difference between group 0 and group 1. There was a significant difference between group 0 and group 2+. A significant difference was also seen between group 1 and group 2+. *P* values less than 0.05 were defined as significant

There was a weak negative correlation between CCI score and JOA improvement rate (*r* = − 0.20.) (Fig. 1). However, there was no relationship between CCI and operative time, intraoperative blood loss, LOS, and direct cost (Table 2). The improvement rate of each group was 74.9% (group 0), 74.5% (group 1), and 67.1% (group 2+). There was no significant difference between group 0 and group 1. However, there was a significant difference between group 0 and group 2+ (*P* = 0.002). A significant difference was also seen between group 1 and group 2+ (*P* = 0.009) (Fig. 2). The mean LOS and direct cost of the three groups were 20 days (group 0), 20 days (group 1), and 22 days (group 2+), and $20,637 (group 0), $21,049 (group 1), and $21,899 (Group 2+), respectively. A significant difference between LOS and direct cost was only recognized between group 0 and group 2+ (Table 3).

**Table 2:** The correlation coefficient between CCI score and each item

	Correlation coefficient with CCI
Operative time	− 0.01
Intraoperative blood loss	− 0.0007
Length of stay	0.18
Direct cost	0.16

**Table 3:** Length of stay and direct cost between the three groups

	Length of stay (day)	Direct cost (US dollar)
Group 0	20.3	20637.6
Group 1	20.9	21049.3
Group 2+	22.3	21899.3
	*P* value	
Group 0–group 1	1	1
Group 0–group 2+	0.009	0.03
Group 1–group 2+	0.05	0.3

## Discussion

As medical technology including medication, equipment and educational programs progresses, surgery will be indicated for a broader patient subset. Elderly patients often have several comorbidities, and younger patients often have cardiovascular disease, have previously undergone surgery, or have renal disease that requires hemodialysis. For these reasons, identifying the preoperative risk factor is important and essential to not only spine surgery but to all surgeries in general. The stratification of comorbidities is helpful for comprehensively understanding the patient’s general condition.

Degenerative lumbar disease including spondylolisthesis and lumbar spinal canal stenosis is a common and well-known pathology. It presents with symptoms that can include low back pain, leg pain, and neurologic deficits that affect daily life. Conservative therapy is usually the first choice for initial management, but if unsuccessful surgical intervention is considered. PLIF is a common general procedure for degenerative lumbar disease. The clinical outcome of PLIFs is widely recognized to be satisfactory [9–11]. Liu reported that a PLIF alone improved clinical satisfaction and decreased complications [9]. Degenerative spondylolisthesis is more common in patients over 60 years of age [15]. As the patients age, they are more likely to develop this disease, and their likelihood of several comorbidities may also be higher.

The impact of comorbidities on spine surgery has been reported, but most prior studies described readmission, mortality, and complications [1, 7, 8, 16, 17]. One of them reported that a higher CCI was associated with increased postoperative complication rates following a minimally invasive transforaminal lumbar interbody fusion [16]. Derman et al. [18] reported that a preoperative CCI of 1 or greater was associated with an increased risk of a subsequent emergency department utilization or a hospital readmission after cervical spine arthrodesis. In contrast, another report concluded that CCI is a useful comorbidity index, but was not completely predictive of the incidence of a major complication [19]. Similarly, adverse complications in this study were equivalently distributed between each group, and not associated with the number of comorbidities.

A few reports have been published about the relationship between comorbidities and clinical outcome [20]. Yagi et al. reported in detail about clinical outcomes and CCI after spine surgery, but conceded that the chief limitation of his work is that different instruments were used to measure clinical outcomes [20]. As a result, they could not compare the clinical outcomes directly.

In this study, the clinical outcome of all patients was evaluated using the JOA score. Clinical outcomes were calculated fairly. We also directly compared these scores with CCI.

There was a weak negative correlation between the JOA improvement rate and CCI in this study. Although we could not identify CCI as a predictor of a poor clinical outcome, this study can identify the negative influence of comorbidities on postoperative clinical outcomes after PLIF. Optimal treatment and control of a comorbidity are necessary before surgery and would be more likely to lead to a satisfactory outcome.

Postoperative rehabilitation is essential to a good clinical and functional outcome. The small frequency or lack of postoperative rehabilitation due to several comorbidities might be related to poor clinical outcomes. In fact, a report in the field of ischemic strokes concluded that a higher modified CCI for stroke score was an independent predictor of poor rehabilitation success, and the authors noted the importance of comorbidities when planning rehabilitation [21]. This conclusion may be similarly applicable to spine. Medical comorbidity was found to be a significant predictor of rehabilitation efficiency in geriatric patients by Patrick [22]. Not only a successful spine surgery but also optimal treatment of comorbidities is necessary for a good clinical outcome. Appropriately treating medical comorbidities may facilitate postoperative rehabilitation and lead to a good recovery after spine surgery.

Every correlation coefficient between CCI score and operative time, intraoperative blood loss, LOS, and direct cost was very weak. However, a higher CCI score (group 2+) was associated with a significantly longer OR time and a more expensive cost of treatment than those in group 0. In contrast to the present work, previous research studies concluded that a greater comorbidity burden as reflected by a higher CCI did not lead to a prolonged hospital stay or an increased direct cost [17]. The different way in which we divided groups by CCI score vs. previous research methodologies might have influenced these results.

The findings of the present study are useful and beneficial not only for spine surgeon but for patients as well. These results help surgeons to indicate a patient for surgical intervention using a PLIF, and to obtain informed consent from the patients before surgery. Preoperative comorbidities and expected clinical outcomes must be discussed with patients because they can recognize and understand their own status. Furthermore, this information helps them to decide whether they will undergo spinal surgery at all based on if it can render a satisfactory outcome.

This study has several limitations. First, its sample size is small because it is a single-center study.

A larger patient sample may achieve a more robust correlation between CCI and postoperative clinical outcome. Secondary, this study did not take into consideration the age of the patients. Several prior works added a 0–4 weighted score depending on the age to the original CCI score [16, 17]. Patient age is an important surgical factor and is related to comorbidity. It might influence the CCI score and its correlation with clinical outcomes in this study. Patient age will be considered in further studies for more accurate results. Lastly, the individual cost for a comorbidity is unknown. Direct cost included all hospital charges. It was difficult to isolate the cost of a comorbidity, such as medication or treatment and compare it between comorbidities. Further research must be done to define the influence of CCI on PLIF outcomes more clearly.

## Conclusions

This is the first effort to evaluate the relationship between CCI and clinical outcomes after PLIF, which is a common, well-known, and widely utilized procedure. The collected data from this study demonstrates several findings. CCI score was weakly correlated with clinical outcomes. The recovery rate of patients with two or more comorbidities was significantly longer than patients without a comorbidity. The development of calculation tools for comorbidity that include age and surgical measurement should contribute to predict postoperative outcome, and the surgeon must take the patient’s comorbidity status into consideration when planning a surgical intervention in order to achieve a good clinical outcome.

## Data Availability

Not applicable.

## Abbreviations

CCI: Charlson comorbidity index
PLIF: Posterior lumbar interbody fusion
LOS: Length of stay
JOA: Japanese Orthopaedic Association
